# Training health professionals to reduce overreporting of birthing people who use drugs to child welfare

**DOI:** 10.1186/s13722-024-00466-6

**Published:** 2024-04-26

**Authors:** Sarah C. M. Roberts, Kimá Joy Taylor, Karen Alexander, Daisy Goodman, Noelle Martinez, Mishka Terplan

**Affiliations:** 1 Department of Obstetrics, Gynecology, and Reproductive Sciences, University of California, San Francisco, Oakland, CA 94612 USA; 2 Anka Consulting, LLC., Silver Spring, MD USA; 3 Friends Research Institute, 1040 Park Ave., Suite 103, Baltimore, MD 21201 USA; 4 Geisel School of Medicine at Dartmouth, 1 Medical Center Drive, Lebanon, NH 03756 USA; 5 VA San Diego Healthcare System, 3350 La Jolla Village Drive, San Diego, CA 92161 USA

**Keywords:** Opioid, Substance use disorder, Birth history, Parenting, Child welfare

## Abstract

**Background:**

Health care providers are a key source of reports of infants to child welfare related to birthing people’s substance use. Many of these reports are overreports, or reports that exceed what is legally mandated, and reflect racial bias. We developed and evaluated a webinar for health professionals to address overreporting related to birthing people’s substance use.

**Methods:**

This evaluation study collected data from health professionals registering to participate in a professional education webinar about pregnancy, substance use, and child welfare reporting. It collected baseline data upon webinar registration, immediate post-webinar data, and 6 month follow-up data. Differences in both pre-post-and 6 month follow-up data were used to examine changes from before to after the webinars in beliefs, attitudes, and practices related to pregnant and birthing people who use drugs and child welfare reporting.

**Results:**

592 nurses, social workers, physicians, public health professionals, and other health professionals completed the baseline survey. More than half of those completing the baseline survey (n = 307, 52%) completed one or both follow-up surveys. We observed statistically significant changes in five of the eleven opioid attitudes/beliefs and in four of the nine child welfare attitudes/beliefs from baseline to follow-ups, and few changes in “control statements,” i.e. beliefs we did not expect to change based on webinar participation. All of the changes were in the direction of less support for child welfare reporting. In particular, the proportion agreeing with the main evaluation outcome of “I would rather err on the side of overreporting to child welfare than underreporting to child welfare” decreased from 41% at baseline to 28% and 31% post-webinar and at 6-month follow up (p = 0.001). In addition, fewer participants endorsed reporting everyone at the 6 month follow-up than at baseline (12% to 22%) and more participants endorsed reporting no one at the 6-month follow-up than at baseline (28% to 18%), p = 0.013.

**Conclusions:**

Webinars on the legal, scientific, and ethical aspects of reporting that are co-developed with people with lived experience may be a path to reducing health professional overreporting to child welfare related to birthing people’s substance use.

## Introduction

Health care providers are a significant source of child welfare reports related to birthing people’s substance use. Previous work has highlighted the individual, interpersonal, hospital-level, and policy-level factors that influence providers’ decision-making about this reporting [1]. A key policy relevant to this reporting is the Child Abuse Prevention and Treatment Act (CAPTA), a federal law first enacted in 1974, which provides funding and guidance regarding prevention, investigation, and intervention of child abuse and neglect. Although CAPTA did not originally include reporting requirements related to pregnant and birthing people’s substance use, subsequent CAPTA reauthorizations (primarily beginning with the Keeping Children and Families Safe Act of 2003) introduced reporting requirements related to pregnant and birthing people’s substance use [2]. In the past 2 decades, in parallel with the increase in opioid use, [3] state governments have adopted a range of reporting requirements, with the number of states that have one or more child welfare reporting requirements related to pregnant or birthing people’s drug use increasing from 11 in 2000 to 26 in 2021 [4].

During this same time period, the rates of infants investigated by child welfare have doubled, with investigations following reports by medical professionals increasing more rapidly than investigations following reports by other mandatory reporters [5]. More than one-third of these investigations of infants relate to birthing people’s substance use [5]. The racial inequities in these investigations are stark, with 5.4% of Black, 3.2% of Indigenous, and 2.5% of white infants investigated [5].

These reports have significant negative consequences for women, other birthing people, children, and families. Research has found that these reports lead pregnant and parenting people who use drugs to mistrust health care providers and thus avoid and disengage from health care and treatment, including avoiding taking medications for opioid use disorder, [6–11] in turn increasing risk for adverse maternal morbidity and mortality and adverse infant health outcomes. Statutes that mandate child welfare reporting related to pregnant people’s substance use are not associated with decreases in neonatal abstinence syndrome, preterm delivery, low birth weight, infant morbidities, or with infant maltreatment, and do not increase prenatal care utilization or substance use disorder treatment admissions [12–15]. These reports by health care providers may also be a key pathway through which pregnant and birthing people come to the attention of criminal law enforcement agencies who then prosecute them related to drug use during pregnancy [16].

While some of these reports are legally mandated, many are not and consist of “overreports” to child welfare agencies—or reports that go beyond what is legally mandated, e.g. reporting a birthing person based solely on having a history of a substance use disorder or receiving treatment for a substance use disorder [17, 18]. Some reasons for these overreports include limited knowledge among health care providers about what the laws actually require them to report and about the history and current practices/functions of the child welfare system [1]. We thus developed and evaluated a webinar for health professionals to address the problem of overreporting related to birthing people’s substance use. In this manuscript, we describe changes in attitudes/beliefs and behaviors relevant to child welfare reporting of pregnant/birthing people who use drugs, from before to after webinar participation.

## Methods

### Overall study design

This evaluation study collected data from health professionals who registered to participate in a professional education webinar about child welfare reporting. Data were collected upon webinar registration (baseline), immediately post-webinar, and then 6 months later (follow-up). Differences in baseline, post-webinar, and 6 month follow-up data were used to examine changes from before to after the webinars in beliefs, attitudes, and practices related to pregnant and birthing people who use drugs in general and who have opioid use disorders (OUD) in particular, and child welfare reporting. The immediate post-webinar surveys also collected participants’ feedback on the webinars.

### Webinar description

The webinar is one component of a larger project (Doing Right at Birth) that convened experts in health, policy, law, and advocacy, along with people affected by the child welfare system (CWS) to co-develop, implement, and evaluate trainings (webinars, videos, toolkit) for healthcare providers on legal, scientific, and ethical aspects of CWS reporting and consequences for birthing people with OUD. The goal of the overarching Doing Right at Birth (DRB) project is to make interactions between birthing people with OUD and both health care providers and the CWS more ethically sound, respectful, grounded in evidence, and within but not exceeding legal requirements, and thereby to reduce stigma and discrimination and improve treatment engagement and recovery postpartum and beyond. DRB also seeks to address the racial inequities in CWS reporting [19]. DRB is guided by two core values: First, people who use drugs should be treated with dignity and respect when they seek healthcare. Second, parenting is hard, and we support non-punitive approaches that empower the parent, infant, dyad, and family to thrive together. In this manuscript, we focus on one component of DRB—the webinar.

We co-developed webinar content collaboratively with input and contributions from physicians, nurse midwives, public health researchers, nurses, lawyers, social workers, and people with lived experience of CWS involvement, some of whom participated in the process as members of a community advisory board (CAB). The CAB, which met quarterly by videoconference for the duration of the project, included nine people with relevant personal and professional experience. CAB members were compensated for meetings and to prepare for, participate in, and provide input and feedback outside of each meeting. CAB members who contributed content to the webinar, including videos sharing their own experiences, received additional compensation. We also drew on findings from the previous implementation science research led by the first author about how clinicians make decisions about reporting pregnant and birthing people who use alcohol and/or drugs to child welfare [1]. The webinar was approximately 90 min in length and included didactic presentations by physicians and lawyers which we interspersed with brief recorded videos from community advisory board members and others with relevant lived experience. Topics covered included: the history of the CWS, the legal parameters for reporting, the effects of substance exposure on child development, racial inequities in the CWS, and the consequences of CWS reports to pregnant people, their children, families, and communities (Table 1). We also kept the chat feature open so webinar participants could communicate with webinar panelists and each other in real time.

**Table 1:** Webinar content outline

1. *Definitions, values, and DRB project goals*, including defining the term “overreporting” in the context of child welfare reporting 2. *Supporting positive equitable outcomes for birthing people, infants, families, and communities*, including describing recovery-friendly pediatric care, the Healthy People 2030 goals relevant to caring for birthing people who use drugs, and the full continuum of evidence-informed culturally and linguistically effective health and social services for pregnant and birthing people who use drugs 3. *History of the U.S. CWS*, including the historical roots of the CWS in slavery and American Indian residential schools, racial inequities in the CWS, and the way the war on drugs has contributed to the growth of the CWS 4. *CAPTA and what the law says about when reporting is mandated*, including how what the law actually says contrasts with what health professionals often believe laws to require in terms of CWS reporting 5. *Descriptions of what happens after a CWS report is made*, including the focus on investigations and mandated services 6. *Urine drug testing*, including what information it does versus does not provide, and the ways it contributes to overreporting, particularly of Black and Indigenous people 7. *Health professional motivations for CWS reporting*, and how beliefs underlying these motivations relates to existing evidence 8. *Research evidence regarding effects of CWS reporting*, including the lack of evidence that child welfare reporting requirements improve infant health and the lack of evidence consistently linking pregnant people’s drug use to later child maltreatment 9. *Strategies for making change*, including actions health professionals can take to make changes within their hospital, their community, and at the state or federal level	

We delivered the webinar three separate times over a four month period. We conducted preliminary analyses to examine changes in some beliefs/attitudes items from pre- to immediate post-webinar surveys after each of the first two webinars to assess whether changes in attitudes and beliefs were occurring and, if so, if they were in the hoped for direction. We also used information from open-ended responses about feedback on the webinar from the immediate post-webinar surveys to identify areas for improvement in subsequent webinar deliveries. Based on preliminary analyses of pre-post attitudes/beliefs items, which suggested changes in some of the attitudes/beliefs items in the hoped for direction, we did not alter the content of the webinars. Based on open-ended response feedback and the real-time reactions and conversations in the chat, we made some changes in webinar logistics and in how participants’ experienced the webinar. Logistical changes included tightening sections for length and improving audio. Participant experience changes involved adapting the way we framed content, including shifting the wording we used to describe the webinar purpose and content; adding some language to name emotions that might be coming up for people in response to webinar content; and adapting our approaches to managing the chat.

### Study participants and data collection

We advertised the webinar widely through professional networks, our own and others’ listservs, and through social media. 1279 people registered to participate in one of the three webinars. People who registered were invited to participate in the evaluation. Those interested in participating then completed eligibility screening. People were eligible if they worked: with pregnant or birthing people; with newborns; and/or on programs, policies, protocols, or systems related to pregnant or birthing people. Eligible participants then reviewed electronic consent materials, and those who consented were then able to begin the baseline survey. After each of the three webinars, we sent the post-webinar survey at the end of the webinar to participants who had registered for that session and asked them to complete it. We sent the 6-month follow-up survey 6 months after the post-webinar survey and asked participants to complete it. We sent reminders to complete post-webinar and 6-month follow-up surveys three times over the next week after each initial request to complete that survey. Participants were remunerated with a $10 gift card upon completing the post-webinar survey and $50 gift card upon completing the 6 month follow up.

### Measures

*Outcomes*: We included attitudes/beliefs measures in four domains; two (*opioid beliefs* and *child welfare beliefs*) that were central foci of the webinar, one (*urine testing beliefs*) that was a minor focus of the webinar, and one (*general attitude/beliefs or* “*control statements”*) that included items we did not address in the webinar and did not expect would change after webinar participation. These beliefs items were on 7-point Likert scales ranging from strongly disagree to strongly agree, with neither agree nor disagree as the middle of the scale. While we focus on individual items in our analyses, we did look at internal consistency for each domain, using Cronbach’s alpha. Alpha for opioid beliefs was 0.83 (11 items), for child welfare beliefs was 0.72 (9 items), for *urine testing beliefs* was 0.76 (5 items), for *general beliefs* was 0.66 (8 items).

We also assessed people’s descriptions of their own *professional role* in terms of child welfare reporting. Participants could report multiple roles, including roles such as “I have final say in the reporting decision” and “I can influence reporting decisions that other people are making.” We also assessed *reporting behavior*, by asking participants to choose the option that best describes their usual practice in relation to reporting birthing people who used drugs during pregnancy to child welfare: report everyone, report most people, report a few people, report no one. We asked about their overall reporting practices and then their reporting practices for a range of different substances (e.g. alcohol, cannabis, prescription opioids not taken as prescribed, heroin/fentanyl). At baseline, we asked about their past year usual practice. At the 6-month follow-up, we asked about their past 6-month usual practice. We developed these items drawing from existing scales to measure opioid-related stigma [20, 21] and implementation science domains we sought to target with the webinar, [1, 22] content expertise of project team members, community advisory board input, and feedback from a few health professionals who participated in a pilot of the survey.

At the 6 month follow-up, we also asked participants two open ended questions about what they have done differently related to reporting birthing people’s drug use and child welfare in the past 6 months.

*Participant characteristics*: We also collected data about participant characteristics, all categorical variables. These included *health professional role*; whether they provide *direct patient care*; U.S. *region* in which they practice; *urbanicity* in terms of rural, suburban, urban; *career stage*; *age*; *race/ethnicity*; and *gender*.

### Analysis

Analyses focused on describing participant attitudes/beliefs, professional role descriptions, and practices, including how they varied across baseline, post-webinar, and 6 month follow-up time points. Before the first webinar, we identified one child welfare belief as our main outcome: “I would rather err on the side of overreporting to child welfare than underreporting to child welfare.” At the grant proposal stage, we specified that changes in (self-reported) reporting practices would be a measure of success.

For attitudes and beliefs items, we created dichotomous outcomes of agree versus disagree/neither agree nor disagree. We conducted chi-square tests to assess whether there were statistically significant differences across the three study time points. We assessed whether findings changed if we used mixed effects regressions, and thus accounted for clustering by individual; findings were almost entirely consistent with the chi-square analysis and so we have chosen to report the chi-square analyses. We also checked whether findings changed if restricted the sample to those completing one or more follow-ups. For the reporting practices outcome, the main analysis only includes participants who stated that they provided direct patient care, as we asked this question differently for people not providing direct patient care. For attitudes/beliefs and professional role items, we conducted sensitivity analyses restricting to those providing direct patient care.

Open-ended responses were reviewed by the first and third author. After reading through the responses, the first author grouped them into whether the change described was in the intended direction (e.g. towards less testing/reporting, sharing information from the DRB webinar with others); no change or “not applicable;” not possible to characterize direction of change; change in a non-intended direction (e.g. towards more testing/reporting). The third author then reviewed the coding and identified places of disagreement. The first and third author then discussed any discrepancies and came to consensus about coding. We then identified sample quotations to show the range of changes participants described.

## Results

### Sample description

Of 1279 people who registered for the webinar, 821 initiated the eligibility screener, 706 were eligible, and 654 consented. Based on counts of the maximum participants in each of the three webinars, 510 people attended at least part of one of the webinars. 592 registrants (84% of those who were eligible) completed the first set of attitude items in the baseline survey and thus were considered to have completed the baseline survey and were retained in the analysis sample. More than half of those completing the baseline survey (n = 307, 52%) completed one or both follow-up surveys.

Nurses and social workers each comprised about one-fourth of evaluation participants, while physicians comprised 14% and public health professionals 16%. The remaining participants included people with a range of other roles, including doulas, substance use disorder and mental health treatment providers, attorneys, child welfare workers, harm reduction specialists, and students. Most (two thirds) provided direct patient care. Participants practiced in all regions of the U.S., with more from the West and Northeast than Midwest and South. More than half practiced in urban and one-fourth in rural areas. Participants spanned all career stages and ages, with people early in their careers and in their 30s comprising the biggest proportions (41% and 38% respectively) of the sample. Three-fourths of participants identified as white, with 8% identifying as Black and 8% as Hispanic/Latinx. More than 90% of the sample identified as female. There were few demographic differences between those who completed only the baseline survey and those who completed baseline plus one or both follow-ups. The only statistically significant differences in who completed follow-up were in professional role, with more social workers and fewer “other” role participants, as well as more females and fewer males completing follow-up. (See Table 2).

**Table 2:** Sample characteristics

Characteristic	Total n (%) n = 592	Baseline only n = 285	Baseline + 1 or more follow-up n = 307
Professional role			
Physician	82 (14)	42 (15)	40 (13)
Nurse	144 (24)	70 (25)	74 (24)
Social worker	135 (23)	49 (17)	86 (28)
Public health	95 (16)	47 (16)	48 (16)
Other	136 (23)	77 (27)	59 (19)
Involved in direct patient care			
No	204 (34)	97 (34)	107 (35)
Yes	388 (66)	188 (66)	200 (65)
Region			
West	196 (33)	106 (37)	90 (29)
Midwest	102 (17)	45 (16)	57 (19)
South	88 (15)	43 (15)	45 (15)
Northeast	205 (35)	90 (32)	115 (37)
Urbanicity			
Urban	298 (53)	138 (54)	160 (52)
Rural	143 (25)	60 (23)	83 (27)
Suburban	88 (16)	45 (18)	43 (14)
Other	34 (6)	14 (5)	20 (7)
Career stage			
In training	41 (7)	18 (6)	23 (7)
Early	245 (41)	126 (44)	119 (39)
Mid	185 (31)	87 (31)	98 (32)
Late	120 (20)	53 (19)	67 (22)
Age			
< 30	71 (13)	33 (13)	38 (13)
30s	207 (38)	94 (37)	113 (38)
40s	165 (30)	79 (31)	86 (29)
50s	74 (13)	31 (12)	43 (14)
60s or older	34 (6)	14 (6)	20 (7)
Race/ethnicity			
White	424 (75)	181 (70)	243 (79)
Black	46 (8)	28 (11)	18 (6)
Hispanic/Latinx	45 (8)	25 (10)	20 (7)
Asian	11 (2)	5 (2)	6 (2)
Other race/ethnicity	13 (2)	5 (2)	8 (3)
Multiracial	25 (4)	14 (5)	11 (4)
Gender			
Male	23 (4)	18 (7)	5 (2)
Female	526 (93)	231 (89)	295 (96)
Trans/nonbinary/self-define	16 (3)	10 (4)	6 (2)

Fewer people provided data on personal demographic characteristics as those were at the end of the survey; the people missing on these characteristics did not complete the full baseline survey

### Attitudes/beliefs

At baseline, there was limited variation in most of opioid attitudes/beliefs, with 90% or more or 10% or fewer participants agreeing with six of 11 opioid attitudes/beliefs statements. There was also limited variation in urine drug testing beliefs, with 10% or fewer agreeing with two of the five urine drug testing beliefs. There was more variation in other attitudes/beliefs assessed. In particular, four beliefs around which there was more variation at baseline included that people with OUD can safely care for a newborn (76% agree), whether state laws require health professionals to report every birthing person who has used opioids during their pregnancy to child welfare (39% agree), whether pregnant people’s use of opioids is associated with increased risk of child abuse/neglect (55% agree), and whether reporting to child welfare prevents babies born to people with OUD from being harmed (21% agree). (See Table 3).

**Table 3:** Changes in agreement with attitudes regarding birthing people with opioid use disorder and control statements

		% agree			p-value
		Baseline (n = 592) %	Post-webinar (n = 239) %	6 month follow-up (n = 218) %	
opbel_2	An opioid use disorder is a chronic medical condition like diabetes mellitus	88	91	93	ns
opbel_3	People with opioid use disorders can get well and return to productive lives	98	96	99	ns
opbel_4	People with opioid use disorders can safely care for a newborn	76	82	85	0.013
opbel_5	People taking medication for opioid use disorders can safely care for a newborn	91	94	95	ns
opbel_11	Birthing people with opioid use disorders have the right to parent their children	91	95	95	0.038
opbel_1	Individuals with opioid use disorder only have themselves to blame for their problem	2	3	2	ns
opbel_6	State laws require health professionals to report every birthing person who has used opioids during their pregnancy to child welfare	39	26	29	< 0.001
opbel_7	Pregnant people’s use of opioids is associated with increased risk of child abuse/neglect	55	34	35	< 0.001
opbel_8	Reporting to child welfare prevents babies born to people with opioid use disorder from being harmed	21	13	14	0.003
opbel_9	Newborns born to a birthing person with an opioid use disorder are better off in foster care than remaining with the birthing person	3	0	4	ns
opbel_10	A birthing person who is receiving methadone or buprenorphine for opioid use disorder should have their newborn removed from their care	3	2	2	ns
cwbel_1	Healthcare workers are more likely to report Black and Indigenous newborns than white newborns to child welfare related to a birthing person’s drug use	77	82	82	ns
cwbel_4	Fear of being reported to child welfare keeps pregnant and postpartum people who use drugs from using healthcare services	96	95	96	ns
cwbel_2	If a health professional does not report a birthing person who uses drugs to child welfare, the health professional risks getting in trouble	56	45	47	0.006
cwbel_3	Reporting to child welfare connects birthing people who use drugs to important services	54	39	47	< 0.001
cwbel_5	The child welfare system protects children from harm	37	23	34	0.001
cwbel_6	I would rather err on the side of overreporting to child welfare than underreporting to child welfare^a^.	41	28	32	0.001
cwbel_7	I bear the brunt of legal responsibility if something bad happens to a baby after a birthing person who uses drugs and their baby leave the hospital	25	26	18	ns
cwbel_8	Where I work, there is a lot of disagreement about child welfare reporting related to birthing people who use drugs	47	51	44	ns
cwbel_9	I bear the emotional burden if something bad happens to a baby after a birthing person who uses drugs and their baby leave the hospital	53	51	49	ns
utoxbel_1	Drug testing without consent violates patient autonomy	82	85	86	ns
utoxbel_2	Birthing people who refuse a drug test should be reported to child welfare	10	8	7	ns
utoxbel_3	Universal urine drug testing of newborns reduces racial bias in child welfare reporting	48	36	45	0.014
utoxbel_4	Results from a urine drug test during the labor and delivery hospitalization indicate whether a birthing person can safely care for a newborn	7	3	6	ns
utoxbel_5	Drug testing a newborn without parental consent is ethical	22	17	20	ns
genbel_4	Racial differences in birth outcomes are due to systemic racism	84	89	90	ns
genbel_2	I personally know several people who have been diagnosed with a substance use disorder	78	81	80	ns
genbel_1	I feel people with diabetes are at fault for their disease	5	4	3	ns
genbel_3	Racial differences in birth outcomes are due to differences in individual behaviors of pregnant people	13	8	11	ns
genbel_5	The main reason more Black and Indigenous children are reported to child welfare is because those families need more help	4	5	4	ns
genbel_6	In my experience, there are some groups of people who just don’t have the skills to parent successfully	19	22	20	ns
genbel_7	In my experience, there are some groups of people who just don’t have the resources to parent successfully	43	34	33	0.006
genbel_8	I feel more comfortable caring for patients with the same racial/ethnic identity as me	16	15	17	ns

^a^Main outcome

Overall, we observed statistically significant changes in five of 11 opioid attitudes/beliefs and in four of nine child welfare attitudes/beliefs from baseline to follow-ups. We observed few changes in urine drug testing (one of five) or general attitudes/beliefs (i.e. “control statements) (one of eight). (See Table 3).

We observed statistically significant increases in the proportion agreeing that people with OUD can safely care for a newborn and have the right to parent their children. We observed decreases in the proportion agreeing that state laws require health professionals to report every birthing person who has used opioids to child welfare, that pregnant people’s use of opioids is associated with increased risk of child abuse/neglect, and that reporting to child welfare prevents babies born to people with OUD from being harmed. Other than the belief that birthing people with OUD have the right to parent their children, where the proportion agreeing increased from 91 to 95% (p = 0.038), the opioid attitudes/beliefs where no statistically significant changes were observed were those with less variation at baseline (i.e. Opioid beliefs 2, 3, 5, 9, 10, as well as opioid belief 1); this was also the case with one of the child welfare beliefs (Child welfare belief 4), two of the urine drug testing beliefs [2 and 4], and two of the general beliefs (1 and 5).

Regarding child welfare beliefs, the proportion agreeing with the main outcome of “I would rather err on the side of overreporting to child welfare than underreporting to child welfare” (child welfare belief 6) decreased from 41% at baseline to 28% and 31% post-webinar and at 6-month follow up respectively (p = 0.001). We also observed changes in beliefs about whether the child welfare system connects birthing people who use drugs to important services and whether the child welfare system protects children from harm (Child welfare beliefs 3 and 5). While fewer agreed at follow-up than baseline that health professionals risk getting in trouble for not reporting to child welfare (Child welfare belief 2), we did not observe statistically significant changes in beliefs related to bearing the emotional or legal burden if something bad happens to a baby after a birthing person who uses drugs and their baby leave the hospital (Child welfare belief 7 and 9).

Of the three urine drug-testing beliefs where there was variation at baseline, we observed one statistically significant change in the proportion agreeing across baseline to follow-ups (proportion agreeing that universal drug testing of newborns reduces racial bias in child welfare reporting) although the change seemed to be present only at the immediate post-webinar follow-up and not at 6 months (48% to 36% to 45%, p = 0.014). We did not observe changes in beliefs as to whether drug testing without consent is ethical.

Overall, we observed only one statistically significant change in general beliefs (“control statements”). This change was in the statement that there are some groups of people who just don’t have the resources to parent successfully (43%, 34%, 33%, p = 0.006).

Sensitivity analyses restricting the sample to just those completing one or more follow-up were substantively similar, with only one statistically significant association no longer significant (opioid belief 11). Sensitivity analyses restricting the sample to just those providing direct patient care were substantively similar, with one statistically significant association no longer significant (again, opioid belief 11), and two beliefs (child welfare belief 8 and general belief 3) becoming statistically significant.

### Professional role

We observed no statistically significant changes in what participants perceived their professional roles to be related to child welfare reporting. (Insert Table 4) This finding was consistent when we restricted the sample to only those providing direct patient care and also when we restricted the sample to those completing at least one follow-up.

**Table 4:** Changes in professional role

	Baseline (n = 573) %	Post-webinar (n = 237) %	6-month follow-up (n = 216) %	p-value
"I have no role in reporting decisions for individual patients"	29	24	24	ns
"I have final say in the reporting decision"	11	13	16	ns
"I raise questions about whether a report is necessary/appropriate"	52	54	58	ns
"I can influence reporting decisions that other people are making"	39	43	44	ns
"I can influence the outcomes of child welfare reports, if they are made"	22	18	21	ns
"I am/have been involved in efforts to change/improve our hospital policy/protocol related to child welfare reporting for birthing people who use drugs"	30	31	36	ns
"I am/have been involved in efforts to change/improve our state policy related to child welfare reporting for birthing people who use drugs"	20	20	22	ns

### Practice changes

**Fig. 1:**
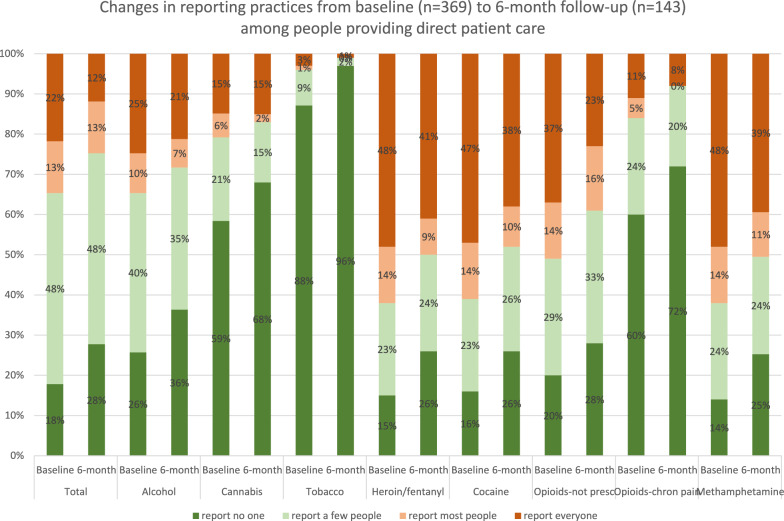
Changes in reporting practices from baseline to 6 month follow-up among people providing direct patient care

Regarding usual practices in relation to reporting birthing people who used drugs during pregnancy to child welfare, fewer participants reported reporting everyone at the 6-month follow-up than at baseline (12% to vs 22%) and more participants reported reporting no one at the 6-month follow-up than at baseline (28% vs 18%), p = 0.013 (See Fig. 1). We observed similar decreases in reporting for all substance-specific reporting practices questions, with the exception of alcohol and cannabis, where the proportions reporting everyone were relatively lower than for other drugs. These findings were robust in sensitivity analyses, except for cannabis-related reporting, where the reduction in reporting was statistically significant in the model restricted to only those completing follow-up.

In response to the open-ended question that asked participants completing the 6 month follow-up to share one or more things they have done differently related to reporting birthing people’s drug use and child welfare in the past 6 months, 83 reported an action in the intended direction (i.e. towards less testing/reporting, sharing information from the DRB webinar). See Table 5 for examples of actions in the intended direction. Forty-three participants described either no change or said “not applicable;” 24 reported more involvement, learning, or reflection on the topic, although it was not possible to characterize any direction of change; and 23 described testing or reporting changes that were in the unintended direction, i.e. towards more testing or more reporting.

**Table 5:** Examples of actions in the intended direction (i.e. towards less testing/reporting and sharing information from the DRB webinar

“Eliminated urine drug screens from our intake” “Shared research with colleagues regarding racial discrimination in urine drug screening” “Only refer to child welfare if there is evidence of abuse, neglect, or maltreatment” “Explore the effect of substance use on a person’s behaviors/resources/abilities, rather than just use the substance use as the outcome of interest” “Challenge status quo and ask supervisor about long standing hospital policies to see if changes can be made or where the wiggle room is” “I have spoken frequently to other staff about our outdated reporting policy as a way to build support for a change to the current reporting policy at my facility”	

### Policy changes

Close to 15% of participants reported that their hospital policy related to urine drug testing and/or related to child welfare reporting changed in the past 6 months. Of the 25 providing information about policy changes, most [18] described the policy changing to support less urine drug testing and/or less reporting, or adding consent before drug testing. For others, it was not clear what direction the change was in, e.g. “we changed some ‘risk factors’ around prenatal care. We came to a consensus on what ‘affected’ means,” or what the change was, e.g. “currently being revised.”

## Discussion

This webinar was part of the Doing Right at Birth project, which has a goal of reducing overreporting to the CWS related to birthing people’s drug use. We anticipated that participating in the webinar would be associated with changed attitudes/beliefs and practices related to CWS reporting. Evaluation findings suggest that webinars were associated with changes in key child welfare and opioid attitudes/beliefs, while having little association with “control statements.” The observed changes were in the hoped for direction of reducing overreporting to the CWS. Findings also suggest that webinars were associated with decreased child welfare reporting of birthing people who use drugs. We did not identify any changes in attitudes/beliefs or reporting practices that went in the opposite direction of what we intended. Further, while some webinar participants did not report doing anything differently related to birthing people’s drug use and child welfare over the six months after they participated in the webinar, most of those who described a change described a change in the direction of reduced urine drug testing and reduced reporting. While some participants did report hospital policy changes, attributing them to webinar participation seems like a stretch, given how long it takes to make policy changes.

Despite the key changes from before to after the webinars, it is also important to note that, even after webinar participation, significant proportions of participants still agreed with statements that are likely barriers to reducing overreporting, [1] e.g. that reporting to child welfare connects birthing people who use drugs to important services and protects children from harm and that they bear the emotional burden if something bad happens to a baby after a birthing person who uses drugs and their baby leave the hospital. While we specifically chose some attitudes/beliefs related to explicit and more implicit racism as our control statements and thus did not expect them to change, it is also important to note that one in five and one in three participants did endorse some of the beliefs we included as indicators of implicit racism—i.e. that some groups of people just don’t have the skills to parent successfully. We also note, though, the almost complete level of agreement with some attitudes/beliefs related to general opioid-related stigma—e.g. that OUD is a chronic medical condition like diabetes and that people with OUD can get well and return to productive lives [20]—perhaps is result of other efforts or an indicator of who registered for the webinars.

It is also important to note where we did not observe changes where we hoped we might have—e.g. in attitudes/beliefs regarding racially inequitable reporting, bearing the emotional and legal burden if something bad happens to the baby, and urine drug testing more broadly. While the drug testing and emotions attitudes/beliefs were less of the focus of webinar, that is unlikely to be the only reason these beliefs did not change. As emotions related to caring for birthing people who use drugs and the reporting decision can be intense and urine drug testing is ingrained in hospital protocols and practices [1], additional efforts to intervene on these attitudes/beliefs may be important. However, if policy and practice changes are occurring, it is possible that people may have new and different emotional experiences caring for birthing people who use drugs in a different policy context, a question worth investigating in the future.

There are a number of limitations to note. First, we do not know whether it was the webinar participation itself or the choice to register for the webinar that contributed to changes in attitudes and practices. It is possible that those who registered for the webinar may have already been more open than other health professionals to changing their attitudes and practices. We also do not have information about which participants actually watched the entire webinar. Second, reporting practices were self-reported. We do not know how they match with actual practices. Third, we did have loss to follow-up from the baseline to follow-up surveys. However, methods that account for loss to follow-up and restricting analyses to just those completing both baseline and follow-up did not have substantive differences.

## Conclusions

Webinars on the legal, scientific, and ethical aspects of reporting co-developed with experts in health care, public health, and law along with people with lived experience may be helpful in reducing health professional overreporting to child welfare related to birthing people’s substance use and could be an important component of a multipronged approach to reducing stigma.

## Data Availability

Datasets analyzed during the current study are available from the corresponding author on reasonable request.

## Abbreviations

CAPTA: Child abuse prevention and treatment act
OUD: Opioid use disorder
CWS: Child welfare system
DRB: Doing right at birth
